# The emerging role of LRRK2 in tauopathies

**DOI:** 10.1042/CS20220067

**Published:** 2022-07-11

**Authors:** Susanne Herbst, Patrick A. Lewis, Huw R. Morris

**Affiliations:** 1 Department of Comparative Biomedical Sciences, Royal Veterinary College, University of London, London, U.K.; 2 Department of Neurodegenerative Disease, UCL Queen Square Institute of Neurology, London, U.K.; 3 Aligning Science Across Parkinson’s (ASAP) Collaborative Research Network, Chevy Chase, MD 20815, U.S.A.; 4 Department of Clinical and Movement Neuroscience, UCL Queen Square Institute of Neurology, London, U.K.

**Keywords:** LRRK2, Parkinson's disease, Progressive Supranuclear Palsy, Tau

## Abstract

Parkinson’s disease (PD) is conventionally described as an α-synuclein aggregation disorder, defined by Lewy bodies and neurites, and mutations in leucine-rich repeat kinase 2 (LRRK2) are the most common autosomal dominant cause of PD. However, LRRK2 mutations may be associated with diverse pathologies in patients with Parkinson’s syndrome including tau pathology resembling progressive supranuclear palsy (PSP). The recent discovery that variation at the LRRK2 locus is associated with the progression of PSP highlights the potential importance of LRRK2 in tauopathies. Here, we review the emerging evidence and discuss the potential impact of LRRK2 dysfunction on tau aggregation, lysosomal function, and endocytosis and exocytosis.

## Introduction

The primary focus of neurogenetics has been the discovery of causative disease genes, first as rare pathogenic variants in familial disease, and then common risk variants in case control studies. These discoveries show us that disease loci may be pleiotropic, that is, there are a range of pathogenic mechanisms at single disease loci. For example, the SNCA locus (encoding α-synuclein) can contain rare copy number variants (duplication and triplications) and rare coding mutations, which segregate with autosomal dominant Parkinson’s disease (PD) but also common risk variants, identified in large-scale case–control studies. Although the mode of inheritance and penetrance varies, in this example PD may be caused by a common pathological mechanism, as different mechanisms may lead to increased transcription of α-synuclein causing protein aggregation and neurodegeneration [1].

In clinical research, the focus has been on modifying the clinical disease course, that is, on developing treatments that will slow or halt the progression of the disease. This might relate to delay in the age at onset, involving a modification of the preclinical disease process before diagnosis, or a delay in the trajectory/progression of disease after diagnosis. Current disease-modifying treatment trials across neurodegenerative disease seek to intervene in patients with early clinical disease. The integration of the recent advances in the neurogenetics of causative gene discovery with new treatment modalities, such as monoclonal antibody and antisense oligonucleotide (ASO) therapy, holds much promise. Gene-directed therapy has largely focused on risk genes. There has been much less focus on the discovery and treatment of modifying factors, that is, factors that change disease progression after diagnosis.

Leucine-rich repeat kinase 2 (LRRK2) is a protein that is primarily linked with PD based on rare coding mutations that segregate in autosomal dominant families (e.g., G2019S, N1437D), coding variants identified through case/control analysis (e.g., G2385R), and common (noncoding) variation associated with clinically diagnosed PD identified in large genome-wide association studies [2]. Recently, it has been shown that the association between common variation at the LRRK2 locus and PD risk may be mediated by an effect on increased LRRK2 expression in a subpopulation of microglia [3]. LRRK2 is a complex protein that comprises several protein–protein interaction domains, a GTPase domain, and a kinase domain. Recent genetic, pathological, cell, and animal model-based research has highlighted diverse LRRK2 functions, with a variety of genetic mechanisms, which can be both a cause and modulator of disease. PD is defined as a synucleinopathy, with abnormal α-synuclein deposits in Lewy bodies and Lewy neurites. However, from the earliest stages of LRRK2 research, there has been evidence that LRRK2 may be important in the pathogenesis of other neurodegenerative pathologies, as LRRK2-PD does not always present with Lewy body pathology [4,5]. More recently, LRRK2 was highlighted as a genetic contributor to disease progression in the primary tauopathy progressive supranuclear palsy (PSP). PSP belongs to a group of neurodegenerative diseases characterized by the accumulation of abnormal forms of the microtubule-associated protein tau, commonly referred to as the tauopathies, in different cells and regions of the brain [6].

Here, we review the recent research particularly with respect to LRRK2 in tauopathies, and highlight important questions that need to be addressed as we advance toward disease-modifying therapies as there is emerging evidence that LRRK2 may be a therapeutic target beyond PD.

## Evidence for tau pathology in LRRK2 PD

The LRRK2 locus was originally identified through linkage as PARK8 on chromosome 12p12 by Funayama et al. [7] in a kindred with autosomal dominant parkinsonism. The family was subsequently shown to carry the pathogenic LRRK2 I2020T mutation. In this family, the LRRK2 mutation shows incomplete penetrance and the predominant pathology is pure nigral degeneration without Lewy body or neurofibrillary tangle formation [8,9]. Since the original description of the PARK8 kindred, eight mutations have been described as autosomal dominant mutations: N1437H/D, R1441C/G/H, Y1699C, G2019S, and I2020T in exons 31, 35, and 41 of the LRRK2 gene [10]. There are marked regional differences in the prevalence of autosomal dominant LRRK2 mutations with N1437D occurring in Chinese populations, R1441G in Spanish/Basque populations, G2019S in North African/Jewish/Northern European populations, and I2020T in Japanese patients.

In a review of the pathology of PD cases with LRRK2 mutations published in 2015, the majority of reported pathological cases with recognized autosomal dominant LRRK2 mutations did not have Lewy body pathology [4]. Notably, Asian cases were particularly likely to have non-Lewy body pathology, but they are also more likely to have the I2020T mutation, so it is difficult to distinguish the effect of background genetics from the effects of different LRRK2 mutations. The absence of Lewy body pathology does not exclude the presence of toxic oligomeric α-synuclein species, but due to limited data available, the presence of other immunoreactive α-synuclein inclusions has thus far not been systematically assessed in LRRK2 mutation carriers.

It is of interest that neurofibrillary tangles, which are composed of aggregates of hyperphosphorylated tau, were noted in 5/37 (14%) of LRRK2 mutation patients and were found in both Y1699C and G2019S cases. However, the I2020T cases showed no recognized protein aggregate pathology. The systematic review of the pathology associated with LRRK2 mutations referred to Alzheimer-like pathology of Braak stage >3, but further characterization of the morphology and isoform composition of tau was not reported [4].

## Evidence for a role of LRRK2 in tauopathies

There have also been a number of case reports of individuals with autosomal dominant LRRK2 mutations with clinical and pathological features of PSP. PSP is a rapidly progressing tauopathy, which has been classified into subtypes, including classic PSP-Richardson syndrome, PSP-parkinsonism, and PSP-progressive gait freezing. PSP-Richardson syndrome presents with early postural instability, bulbar dysfunction, and dementia, and patients have a mean survival of 6–9 years [11]. The classical form of PSP, PSP-Richardson syndrome has a prevalence of 6/100,000 representing about 5% of parkinsonism cases in the United Kingdom [12]. Other PSP subtypes show a slower rate of progression. The presence of neurofibrillary tangles, consisting of hyperphosphorylated tau, in tufted astrocytes and neurons in subcortical and cortical brain regions are a pathologic hallmarks of the disease.

In many cases, LRRK2 mutations associated with PSP pathology have occurred in families where other individuals with the same mutation have typical PD and Lewy body pathology. One of the original families linked to the LRRK2 locus (“Family D”) carrying the LRRK2 R1441C mutation contained one individual with PSP-type neurofibrillary tangles [5]. A family was described in Crete where an individual initially presented with PD at the age of 61 years, but 8 years later developed typical features of PSP with bulbar failure, falls and a vertical supranuclear gaze palsy. This individual carried the LRRK2 R1441H mutation [13]. Although pathological confirmation is lacking, it appears that the initial presentation was with PSP-Parkinsonism, which evolved to a PSP-Richardson syndrome phenotype.

A systematic study of 1039 pathologically confirmed PSP cases and 145 cases of corticobasal degeneration (CBD), which is also classified as a tauopathy, identified two recognized pathogenic LRRK2 mutations (G2019S and R1441C), and two mutations that were predicted to be pathogenic (A1413T and R1707K). Therefore, the prevalence of putative autosomal dominant mutations in LRRK2 in PSP can be estimated to be 2–4/1184 (0.17–0.34%). Only one of the four cases were reported to have an autosomal dominant family history [14].

There is variation in the tau pathology reported in association with LRRK2 mutations. The Mayo case series reported a G2019S case with typical PSP features, with tufted astrocytes and involvement of the subthalamic nucleus and globus pallidus, whereas an earlier detailed report of the Canadian family SK reported a 4-repeat predominant tauopathy in a G2019S patient but with a different tau distribution to PSP without involvement of the globus pallidus or subthalamic nucleus [15]. Some investigators have highlighted Alzheimer’s disease (AD)-type tau in LRRK2 mutation cases. A recent study of 11 LRRK2 mutation carriers showed that all had tau pathology to some degree and that this was usually AD-type tau [16].

Genome-wide association studies have not highlighted LRRK2 as a contributor to PSP risk [17–19]. We recently reported evidence for LRRK2 as a genetic modifier of the rate of PSP progression in a genome-wide survival study [20]. In a pooled analysis of 1239 cases, we identified an association between the rs2242367 allele and survival in PSP (hazard ratio: 1.37, *P*=1.38 × 10^−10^). This survival signal for a primary tauopathy is 190 kb from the PD LRRK2 association SNP rs76904798. These association signals appear to be separate as there is no association between rs76904798 and PSP survival, and survival analysis conditioned on the rs2242367 allele does not identify any independent associations at the LRRK2 locus. Colocalization analysis of genetic variants controlling gene expression in whole blood or brain does not show colocalization between the PSP survival variant and the expression level of LRRK2; however, there is a strong colocalization between the expression of a noncoding RNA LINC02555 and PSP survival. Although further cell- and brain-specific studies are needed, LINC02555 could control LRRK2 expression in specific cell types or states in the brain.

## Molecular LRRK2 functions that could contribute to tau pathology

**Figure 1:**
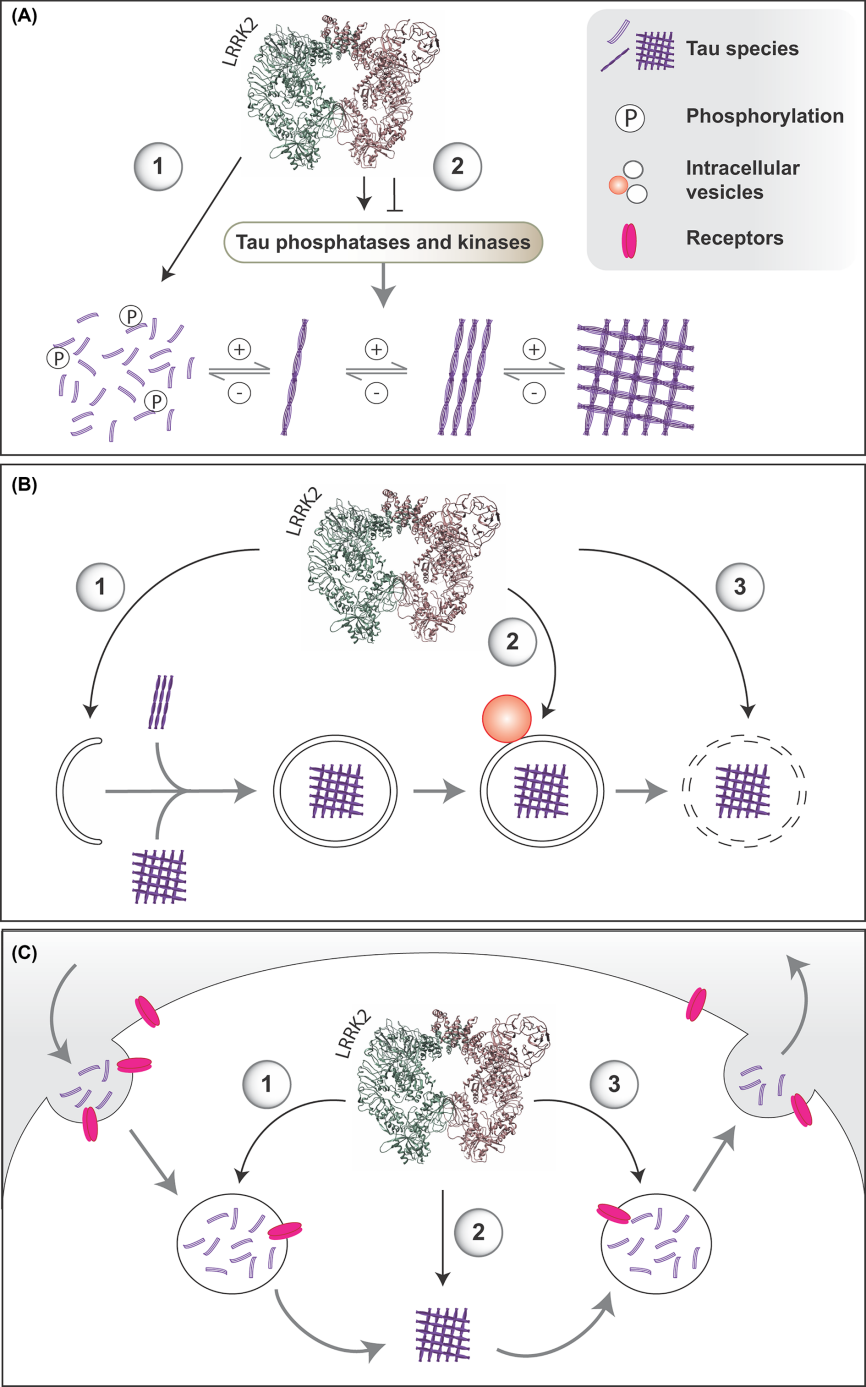
Potential mechanisms by which LRRK2 could impact the development and progression of tau pathology (**A**) LRRK2 could impact **tau aggregation** by phosphorylating tau directly, by binding to microtubules or by altering tau kinase or phosphatase activity. (**B**) LRRK2 affects **lysosomal function** by (1) impairing autophagosome biogenesis, (2) lysosomal fusion, or (3) lysosomal degradative capacity, thereby promoting tau cytoplasmic accumulation, aggregation, and spreading. (**C**) LRRK2 impacts on both **endocytic and exocytic pathways**, which could impact the recycling of tau receptors, or directly affect the intracellular trafficking of tau. Impaired intracellular trafficking could result in (1) decreased tau uptake, (2) increased cytoplasmic accumulation and aggregation, or (3) increased exocytosis. Structure of LRRK2 derived from PDB 7LHT using UCSF ChimeraX.

LRRK2 has been implicated in a wide variety of cellular pathways, ranging from direct binding of microtubules, to uptake and secretion mechanisms such as endocytosis and exocytosis, to playing a role in lysosomal function, thereby having a pleiotropic effect on degradative pathways such as autophagy and inflammation. Tauopathies are characterized by the intracellular accumulation of tau aggregates but it has been suggested that the release of tau aggregates into the extracellular environment might contribute to tau spreading by seeding of soluble tau into aggregated forms when taken up by neighboring cells [21]. Overall, it is plausible that LRRK2 promotes tau pathology by directly impacting the normal function or aggregation of tau, by altering tau intracellular degradation, by interfering with tau endocytic and exocytic pathways, or by promoting an inflammatory environment that favors tau aggregation (Figure 1).

### Direct impact of LRRK2 on tau function

Tau function is regulated by post-translational modification, with phosphorylation of tau being the best characterized. Tau can be phosphorylated at multiple sites and tau phosphorylation results in tau dissociation from microtubules, thereby increasing the cytosolic pool [21]. Tau aggregates associated with tauopathies are found to be hyperphosphorylated; however, it is not known if hyperphosphorylation drives aggregation, or is a consequence of the pathological processes that lead to aggregation [22]. Hyperphosphorylation of tau has been reported in some but not all LRRK2 animal models [23–25], but there is only limited evidence that LRRK2 directly phosphorylates tau and thereby affects its propensity to aggregate [23,26]. Kawakami et al. suggested that LRRK2 is able to phosphorylate microtubule-bound tau, but not free monomeric forms of tau. In contrast, Bailey et al. identified epitopes potentially phosphorylated by LRRK2 on free monomeric isoforms of tau, whereas others were not able to detect direct tau phosphorylation by LRRK2 [24]. Alternatively, it has been suggested that LRRK2 affects tau phosphorylation by regulating the activity of tau kinases. LRRK2 has been reported to increase the activity of the tau kinases GSK-3β [27], but block the recruitment of the tau kinase TTBK2 to centrosomal tubulin [28]. As such, it remains plausible that LRRK2 has a direct effect on tau function by altering tau post-translational modification, but further research is needed to strengthen or dispute this hypothesis.

Additionally, LRRK2 has been reported to directly interact with microtubules. This could have downstream consequences on the interaction between tau and microtubules. Accordingly, an altered cytosolic to microtubule-bound tau ratio could increase tau’s propensity to aggregate. In particular, a subset of LRRK2 pathogenic variants can form filaments on microtubules [29,30], which impairs the motility of microtubule-associated motors [31] and therefore makes it likely to impair the binding of tau. However, LRRK2 microtubule-binding is primarily observed in overexpression systems and when cells have been treated with LRRK2-type I kinase inhibitors [31]. It remains unknown if LRRK2 also forms microtubule-associated filaments *in vivo*, and if this is the case how they contribute to pathology. LRRK2 has also been shown to bind to tubulin without forming filaments, thereby altering microtubule dynamics [32], which could constitute an alternative mechanism explaining how direct LRRK2 microtubule interactions could alter tau-microtubule interactions.

### Lysosomal degradation and proteostasis

The effect of LRRK2 on lysosomal function has been extensively studied, especially with regard to the links between lysosomal function and macroautophagy. LRRK2 inhibition was first shown to promote autophagy induction [33], which gave rise to the idea that pathogenic LRRK2 mutations block autophagy pathways and thereby inhibit the clearance of cytosolic protein aggregates, such as tau and α-synuclein. Since then, more general effects of LRRK2 on lysosomal function have been observed. LRRK2 has been shown to alter the proteolytic capacity of lysosomes [34] and affect lysosomal pH [35,36], with cells harboring LRRK2 pathogenic mutations consistently displaying reduced catalytic function and increased lysosomal pH. Importantly, this phenotype can be reversed upon inhibition of LRRK2 kinase activity. LRRK2 is not found on lysosomes at steady-state levels but is recruited in response to lysosomal stress [37–39] implying that LRRK2 lysosomal function plays a role in the cellular stress response. It is of note that large protein aggregates and misfolded proteins, including tau and α-synuclein aggregates induce lysosomal stress [40,41], and it is intriguing to speculate that tau aggregates are capable of inducing lysosomal stress and LRRK2 activation. These findings would place LRRK2 more generally at the nexus of protein aggregate clearance and imply that LRRK2 could promote tau aggregation and spreading by inhibiting aggregate seed clearance.

### Endocytic and exocytic pathways

LRRK2 has been shown to function during the endocytic recycling of multiple receptors including epidermal growth factor receptor [42] and transferrin receptor [43]. A preprint, which screened for modifiers of monomeric and aggregated tau uptake in the presence of functional transferrin endocytosis, identified LRRK2 alongside the known tau receptor LRP1 [44] as a requirement for tau uptake [45]. Knockdown of the tau receptor LRP1 reduced tau spreading in an *in vivo* murine model of tau neuronal spreading [44]. Therefore, it is tempting to speculated that LRRK2 overactivity could promote tau seeding and spreading in a homologous fashion. Although the mechanistic role of LRRK2 during tau uptake remains elusive, the present study potentially creates a direct link between tau trafficking and LRRK2.

Similarly, there is good evidence that LRRK2 affects retrograde trafficking and exocytosis of lysosomal organelles. In response to lysosomal stress, LRRK2 mediates a signalling cascade that recruits adaptors for molecular motors, which in turn mediate the transport of vesicles along microtubules [39,46]. By examining the axonal transport of autolysosomal organelles, a recent study indicated that LRRK2 activity on autolysosomes favors retrograde transport toward the plasma membrane [47]. In accordance, LRRK2 promoted the exocytosis of stressed lysosomes [37] and was required for the exocytosis of lamellar bodies in alveolar epithelial-type 2 cells [48]. Intriguingly, in an animal model of tau pathology the LRRK2 G2019S mutation increased tau neuronal spread without affecting tau phosphorylation [24]. Similarly, a recent study with inoculation of human AD-tau showed that mice with G2019S mutations had increased retrograde spread of tau pathology [49]. Taken together, these findings raise the possibility that LRRK2 could shift the ratio of extracellular versus intracellular tau toward the extracellular environment, thereby promoting tau aggregation, and cell-to-cell seeding. As such, they also imply that LRRK2 more generally could affect the spreading of protein aggregates in neurodegenerative diseases.

### Neuroinflammation and tau aggregation

LRRK2 has repeatedly been recognized as an inflammatory mediator [50], but the role of inflammation in tauopathies such as PSP remains to be fully investigated. Glial activation and neuroinflammation have been observed in PSP patients [51,52] and the degree of neuroinflammation correlates with PSP disease severity and disease progression [53]. In recent years, the NLRP3 inflammasome has emerged as a propagator of both neuroinflammation and protein aggregation [54,55]. The inhibition of the NLRP3 inflammasome prevents tau aggregation and disease progression in a mouse model of tauopathies [56]. LRRK2 has been implicated in inflammasome activation; however, no direct role in NLRP3 inflammasome activation and IL-1β/IL-18 production has been reported so far [57,58]. It is of interest to note, however, that the commonly used NLRP3 inflammasome activator Nigericin activates LRRK2 [59]. Therefore, a role of LRRK2 in promoting tau aggregation by exacerbating inflammatory processes, cannot be excluded.

## Open questions: LRRK2, PD, and PSP

There are a number of open questions relating to our understanding of the role of LRRK2 in tauopathies. It appears that distinct signals at the LRRK2 locus are associated with progression, but not risk, for PSP, and risk, but not progression, for PD [17,20,60–62]. These effects may relate to cell-type specific expression patterns, or heterogeneity in pathology and disease states and understanding the underlying mechanisms will be fundamental in understanding disease mechanisms in PSP itself and validating LRRK2 as a therapeutic target. Furthermore, the mechanism that links the PSP progression variant to LRRK2 is unclear. Currently, this is hypothesized to be due to an effect of LINC02555 on the expression or translation of LRRK2 mRNA in specific cells, altering LRRK2 protein levels. However, experimental validation in cells relevant to the central nervous system is needed. Similarly, if the PSP linked variant results in LRRK2 kinase overactivity and increased Rab phosphorylation needs to be validated and precisely defined. It may be that different patterns of Rab phosphorylation may be relevant to different diseases and understanding this mechanism will likely lead to new therapeutic insights and targets.

## Outlook: LRRK2 inhibition as a disease-modifying treatment in tauopathies

LRRK2 has emerged as a priority drug target in PD due to the prevalence of mutations in familial disease and evidence for LRRK2 kinase overactivation in idiopathic disease [63]. Additionally, the tractability of kinase activity makes LRRK2 suitable for pharmacological modulation. This has resulted in a number of clinical trials for LRRK2 kinase inhibitors, and an antisense oligonucleotide-targeting LRRK2 expression [64] (Table 1). Emerging work from cell, animal, and human genetics studies highlights the potential role of LRRK2 in tau uptake and the spread of tau pathology. Intriguingly, animal work highlights a role for LRRK2 in the spread of AD pathology, but there is no direct human genetic evidence for a role of LRRK2 in AD. There is strong genetic evidence for variation at the LRRK2 locus as being of importance in the progression of PSP. Further work is needed on cell- and state-specific regulation of LRRK2, the regulatory role of lncRNAs and the main cellular processes relevant to the effect of LRRK2 on tau. However, if the LRRK2 therapies in development prove safe and efficacious in PD, and the evidence for the role of LRRK2 in tauopathies continues to develop, there would be very few obstacles to expand LRRK2-targeting therapies to tauopathies and PSP in particular with the overall aim being to prevent tau seeding and spreading and therefore halt or slow disease progression.

**Table 1:** An overview of LRRK2-targeting therapies currently undergoing clinical trials

Compound name	Mode of action	Phase	Clinical trial ID	Developer	Progress
DNL201	Kinase inhibitor	1/1b	NCT04551534 NCT03710707	Denali Therapeutics Inc.	Completed
DNL151/BIIB122	Kinase inhibitor	1/1b	NCT05005338 NCT04056689 NCT04557800	Denali Therapeutics Inc./Biogen	Completed
BIIB094	ASO	1	NCT03976349	Biogen	Recruiting
DNL151/BIIB122	Kinase inhibitor	2b	NCT05348785	Denali Therapeutics Inc./Biogen	Recruiting

Abbreviations: ASO, antisense oligonucleotide.

## Data Availability

No primary data or secondary data analysis is included in the present manuscript.

## Open Access

Open access for the present article was enabled by the participation of University College London in an all-inclusive *Read & Publish* agreement with Portland Press and the Biochemical Society under a transformative agreement with JISC.

## Abbreviations

AD: Alzheimer’s disease
CBD: corticobasal degeneration
LRRK2: leucine-rich repeat kinase 2
PD: Parkinson’s disease
PSP: progressive supranuclear palsy
ASO: antisense oligonucleotide
NLRP3: NOD-, LRR- and pyrin domain-containing protein 3
